# What is the burden of osteomyelitis in Germany? An analysis of inpatient data from 2008 through 2018

**DOI:** 10.1186/s12879-021-06274-6

**Published:** 2021-06-10

**Authors:** Nike Walter, Susanne Baertl, Volker Alt, Markus Rupp

**Affiliations:** 1 Department for Trauma Surgery, University Medical Center Regensburg, Franz-Josef-Strauß-Allee 11, 93053 Regensburg, Germany; 2 Department for Psychosomatic Medicine, University Medical Center Regensburg, Franz-Josef-Strauß-Allee 11, 93053 Regensburg, Germany

**Keywords:** Osteomyelitis, Bone infection, Epidemiology, Nationwide burden, Musculoskeletal disorder

## Abstract

**Background:**

The epidemiology of osteomyelitis in Germany is unknown, which makes it difficult to estimate future demands. Therefore, we aimed to analyse how the numbers of cases have developed over the last decade as a function of osteomyelitis subtype, age group, gender, and anatomical localization.

**Methods:**

Osteomyelitis rates were quantified based on annual ICD-10 diagnosis codes from German medical institutions between 2008 through 2018, provided by the Federal Statistical Office of Germany (Destatis).

**Results:**

Overall osteomyelitis prevalence increased by 10.44% from 15.5 to 16.7 cases per 100,000 inhabitants between 2008 through 2018. Out of 11,340 cases in 2018, 47.6% were diagnosed as chronic, 33.2% as acute and 19.2% as unspecified osteomyelitis. Men were often affected than women with 63.4% of all cases compared to 36.6%. The largest proportion of patients comprised the age group 60–69 years (22.1%), followed by 70–79 years (21.7%). A trend towards more osteomyelitis diagnoses in older patients was observed. Lower extremities were most frequently infected with 73.8% of all cases in 2018 (+ 10.8% change).

**Conclusions:**

Osteomyelitis remains a serious problem for orthopedic and trauma surgery. Prevention methods and interdisciplinary approaches are strongly required.

## Background

Osteomyelitis has accompanied mankind from its very beginning and still remains a difficult to manage challenge in orthopaedic and trauma surgery [1]. Osteomyelitis can occur to a variety of reasons. Hematogenous infection is possible as well as direct inoculation of bacteria to bone, which occurs in open fractures, after surgery or skin breakdown secondary to vascular insufficiency or peripheral neuropathy [2]. Several classifications for osteomyelitis exist such as the one introduced by Waldvogel and colleagues or George Cierny and John Mader. The former includes a temporal distinction between acute and chronic, which is important for the treatment strategy [2, 3]. In acute osteomyelitis, defined by symptoms less than 2 weeks, empirical antibiotic long-term therapy is feasible since establishment of a mature biofilm might not have taken place, which is the hallmark of chronic osteomyelitis. Once formation of biofilm is accomplished, susceptibility to antibiotic therapy is significantly reduced and eradication of infection without surgical treatment is not possible [3].

While there was no curative therapy until modern times, advances in modern medicine have led to the fact that at least osteomyelitis is no longer associated with an almost inevitable death. Milestones for a curative treatment are the development of surgical treatment concepts which focus on infect eradication followed by bone defect reconstruction. The discovery of penicillin and further development of an array of different antibiotics, the use of local antibiotic carriers as well as surgical procedures such as bone transfer introduced by Ilizarov, Masquelet’s membrane induced technique or free flap surgery invented by Harry J Buncke contributed to the progress in orthopaedic and trauma surgery [4–7]. Although joint replacement procedures and surgical fracture treatment with internal fixation devices have become an integral part of modern medicine enhancing patients’ quality of life, they represent an additional risk of bone and joint infection. For instance, rates of developing a posttraumatic infection are reported to be around 1–2% for closed fractures ranging up to exceeding 30% for Gustilo-Anderson type III open tibia fractures despite prevention strategies [8–11]. Depending on injury severity, success rates only vary between 70 and 90% with a recurrence of the disease in 6–9% of the patients [12–14]. Healthcare costs of fracture-related infections were estimated to be approximately 6.5 times higher than in non-infected cases [15]. As incidences of long bone fractures increase, projected numbers of infection complications are expected to rise as well [16]. Additionally, the global prevalence of diabetes is projected to increase up to 7079 individuals per 100,000 by 2030 [17], hence also heightening the risk of osteomyelitis.

To estimate future demands for this potentially coming challenge and to foresee developments which could be influenced by adaption of prevention and therapeutic measures, analysis of trends in osteomyelitis rates are required. However, no analysis of the epidemiology of osteomyelitis for European countries is available.

We have therefore aimed to answer the following questions: (1) How have the numbers of cases developed over the last decade as a function of osteomyelitis subtype and localization? (2) How does age and gender influence the numbers in the observation period?

## Methods

Data consisting of annual ICD-10 diagnosis codes, which were implemented in Germany in January 2000, from German medical institutions between 2008 to 2018 was provided by the Federal Statistical Office of Germany (Destatis). The dataset included only patients who received inpatient treatment. The ICD-10 code “M86.-” was used to identify patients aged 20 years or older diagnosed with osteomyelitis. A detailed breakdown of these data by age group, gender, type of osteomyelitis and anatomical localization was performed. In particular, for composing the subgroup “acute osteomyelitis, the ICD-10 codes “M86.0, M86.1, M86.2” were used, whereas chronic osteomyelitis was determined by the codes “M86.3, M86.4, M86.5, M86.6” and unspecified osteomyelitis by “M86.8, M86.9″. Localization was retrieved by using the codes “-1, shoulder”, “-2, humerus”, “-3, radius and ulna”, and “-4, hand” to compile the upper extremity subgroup and “-5, femur”, “-6, tibia and fibula” and “-7, ankle and foot” for the lower extremity subgroup, respectively. Prevalence rates were calculated based on Germany’s historical population aged 20 years or older provided by Destatis [18]. Here, the number of inhabitants in each of the 16 German federal states was considered by year of birth for each year of the period 2008 to 2018. The deadline of each year was December 31. Data were analyzed using the statistical software SPSS Version 26.0 (IBM, SPSS Inc. Armonk, NY, USA).

## Results

In 2018, a total number of 11,340 osteomyelitis cases in Germany was reported. In comparison to 10,268 cases in 2008, the overall prevalence increased by 10.44% from 15.5 cases per 100,000 inhabitants to 16.7 cases per 100,000 inhabitants. Between 2008 through 2012 total numbers decreased and subsequently rose again with a maximum of cases in the year 2016. (Table 1). The largest proportion of osteomyelitis cases in 2018 comprised chronic cases (47.6%), followed by 33.2% acute cases and 19.2% unspecified cases. The total number of cases diagnosed with acute osteomyelitis increased by 61.8% from 2327 to 3765 between 2008 and 2018. An increasing trend could also be observed regarding chronic osteomyelitis cases, which heightened by 8.4% from 4984 to 5402 cases in total between 2008 and 2018, whereas osteomyelitis cases classified as unspecified decreased by 26.5% from 2957 to 2173 patients (Table 2).

**Table 1:** Historic development of population and osteomyelitis prevalence from 2008 to 2018

Year	Total numbers	Relative to 2008 [%]	German population 20 years or older	Prevalence per 100,000 inhabitants
2008	10,268		66,346,045	15.5
2009	9932	−3.27	66,400,066	15.0
2010	9893	−3.65	66,549,975	14.9
2011	10,053	−2.09	65,398,514	15.4
2012	10,107	−1.57	65,665,069	15.4
2013	10,452	+ 1.79	65,943,867	15.8
2014	10,351	+ 0.81	66,677,665	15.5
2015	10,860	+ 5.77	67,097,676	16.2
2016	11,480	+ 11.8	67,440,230	17.0
2017	11,331	+ 10.35	67,540,025	16.8
2018	11,340	+ 10.44	67,724,921	16.7

**Table 2:** Historic development of osteomyelitis subtypes from 2008 to 2018

Year	Acute osteomyelitis cases (percentage)	Chronic osteomyelitis cases (percentage)	Unspecified osteomyelitis cases (percentage)
2008	2327 (22.7)	4984 (48.5)	2957 (28.8)
2009	2292 (23.1)	4748 (47.8)	2892 (29.1)
2010	2306 (23.3)	4848 (49.0)	2739 (27.7)
2011	2444 (24.3)	4997 (49.7)	2612 (26.0)
2012	2480 (24.5)	4914 (48.6)	2713 (26.8)
2013	2644 (25.3)	4983 (47.7)	2825 (27.0)
2014	2636 (25.5)	4988 (48.2)	2727 (26.3)
2015	2799 (25.8)	5347 (49.2)	2714 (25.0)
2016	3414 (29.7)	5720 (49.8)	2346 (20.4)
2017	3621 (32.0)	5446 (48.1)	2264 (20.0)
2018	3765 (33.2)	5402 (47.6)	2173 (19.2)

**Fig. 1:**
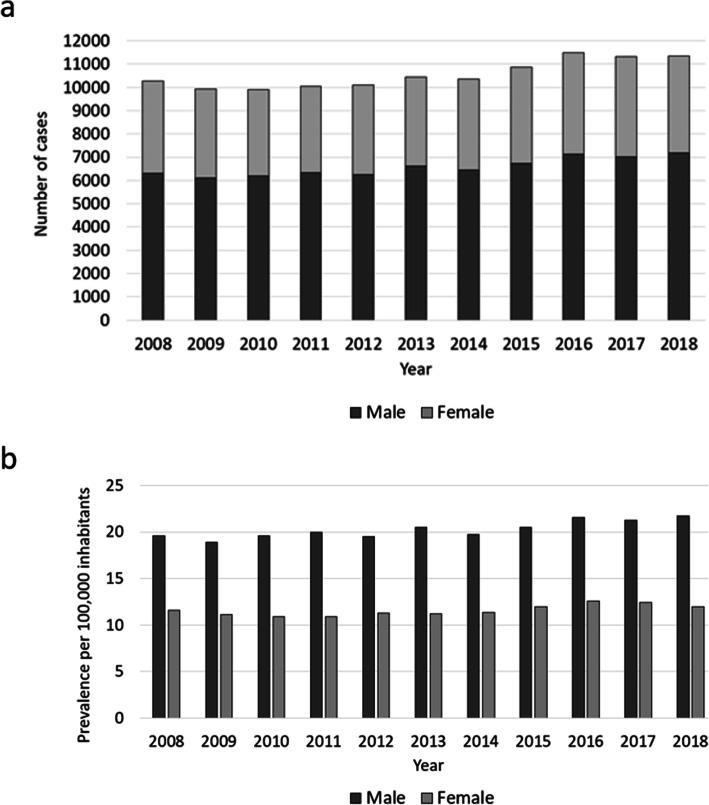
Development of osteomyelitis diagnoses as a factor of gender. **a** Shows the amount of total cases per year. Male cases are shown in dark grey, female cases in light grey. **b** The prevalence per 100,000 male inhabitants is shown in dark grey, prevalence per 100,000 female inhabitants is illustrated in light grey

**Fig. 2:**
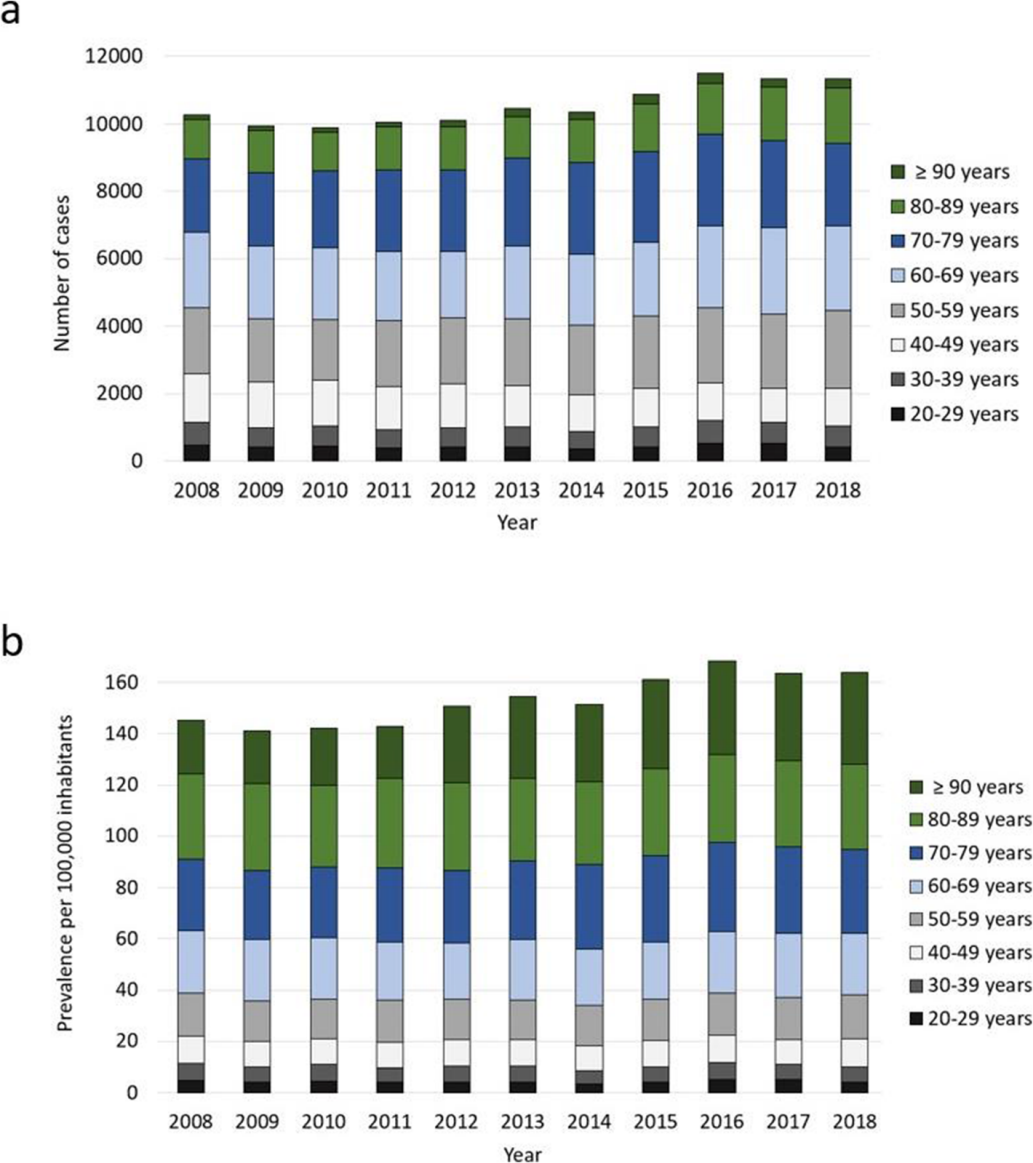
Development of osteomyelitis numbers as a factor of age groups in 10-year increments. **a** Total number of osteomyelitis diagnoses, (**b**) age standardized prevalence per 100,000 inhabitants

**Fig. 3:**
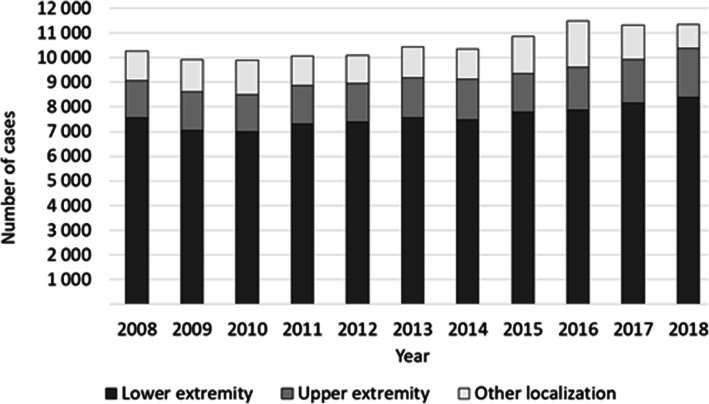
Development of osteomyelitis numbers as a factor of anatomical localization

**Fig. 4:**
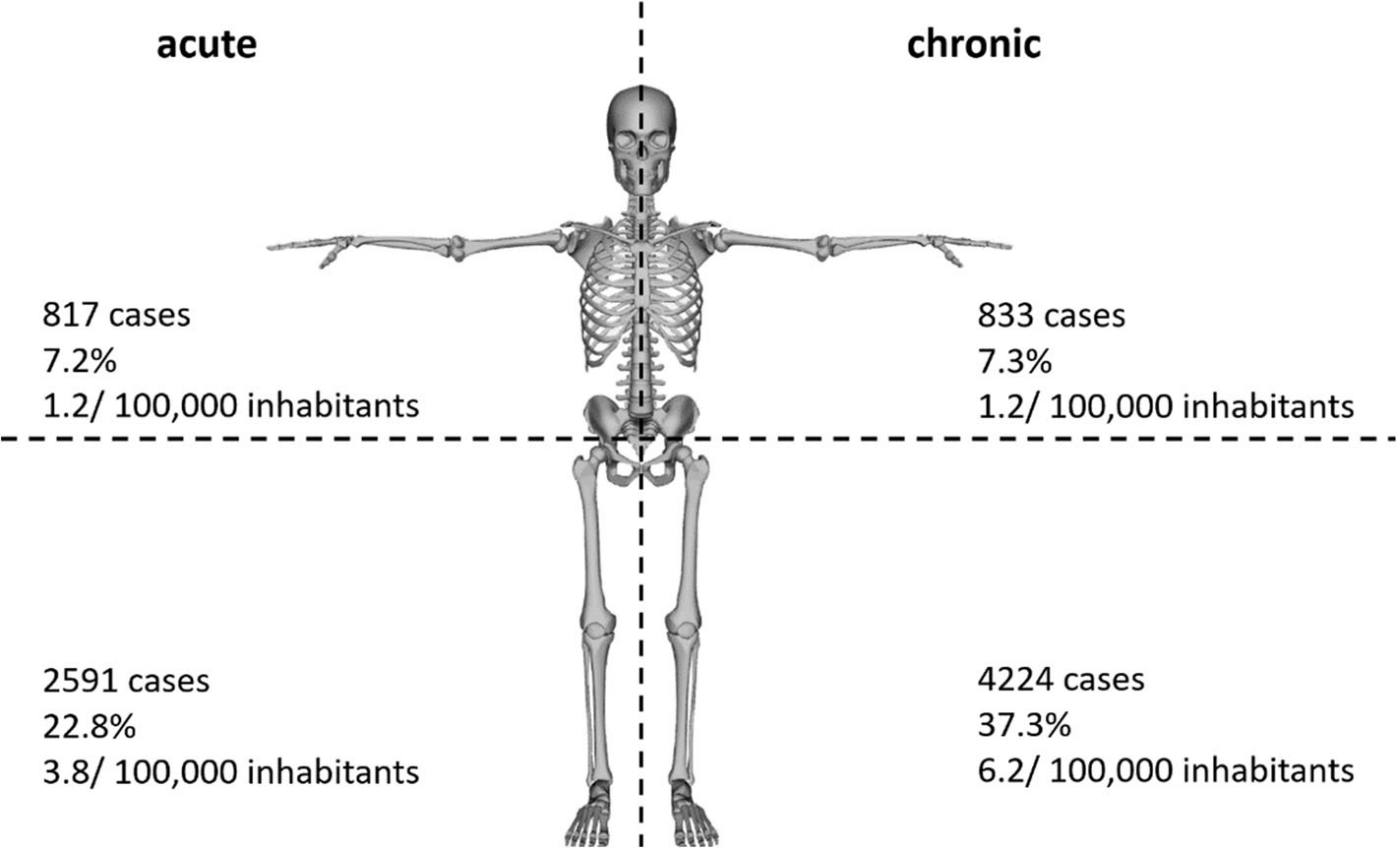
Acute and chronic osteomyelitis cases for the upper and lower extremity, respectively

Overall, men were more often affected than women, whereby the proportion of male cases increased from 61.4 to 63.4% and female cases decreased from 38.6 to 36.6% accordingly (Fig. 1, Table 3). Patients aged 60–69 years comprised the largest cohort with 22.1%, followed by patients aged 70–79 years (21.7%) and patients aged 50–59 years (20.3%). Relative to the year 2008, a trend towards more osteomyelitis diagnoses in older patients can be observed. The largest increase was found in the population aged 90 years or older (+ 115.2% change). In the increment 80–89 years 37.8% more cases were registered and 13.0% more patients in the age between 70 and 79 years were affected. Also, the prevalence heightened in patients aged 60–69 years (+ 11.9% change) and in patients aged 50–59 years (+ 18.4% change). Accordingly, less patients aged 40–49 years were diagnosed with osteomyelitis (− 22.1% change) and numbers decreased in the age group 30–39 years (− 5.9% change) as well as 20–29 years (− 15.4% change) (Fig. 2, Table 3). The most frequently infected region was the lower extremity with 73.8% of all cases in 2018, whereby numbers rose by 10.8% from 7553 cases in 2008 to 8371 cases in 2018. Osteomyelitis occurred in 17.7% of all cases at the upper extremity, with an increase of 32.14% from 1515 cases in 2008 to 2002 cases in 2018. Other regions were involved in 8.5% of all cases, with decreasing prevalence of 19.4% from 1200 cases in 2008 to 967 cases in 2018 (Fig. 3, Fig. 4, Table 3).

**Table 3:** Development from 2008 through 2018 of osteomyelitis cases divided by gender, age and anatomical localization

	2008 n/(%)	2009 n/(%)	2010 n/(%)	2011 n/(%)	2012 n/(%)	2013 n/(%)	2014 n/(%)	2015 n/(%)	2016 n/(%)	2017 n/(%)	2018 n/(%)
Gender											
Male	6304 (61.4)	6121 (61.6)	6192 (62.6)	6349 (63.2)	6259 (61.9)	6612 (63.3)	6455 (62.4)	6733 (62.0)	7126 (62.1)	7028 (62.0)	7189 (63.4)
Female	3964 (38.6)	3811 (38.4)	3701 (37.4)	3704 (36.8)	3848 (38.1)	3840 (36.7)	3896 (37.6)	4127 (38.0)	4354 (37.9)	4303 (38.0)	4151 (36.6)
Age											
20–29 years	467 (4.5)	399 (4.0)	28 (4.3)	384 (3.8)	411 (4.1)	418 (4.0)	360 (3.5)	406 (3.7)	521 (4.5)	505 (4.5)	395 (3.5)
30–39 years	675 (6.6)	587 (5.9)	615 (6.2)	551 (5.5)	580 (5.7)	587 (5.6)	520 (5.0)	588 (5.4)	683 (5.9)	630 (5.6)	635 (5.6)
40–49 years	1452 (14.1)	1360 (13.7)	1340 (13.5)	1271 (12.6)	1291 (12.8)	1224 (11.7)	1093 (10.6)	1169 (10.8)	1113 (9.7)	1011 (8.9)	1131 (10.0)
50–59 years	1944 (18.9)	1866 (18.8)	1818 (18.4)	1967 (19.6)	1958 (20.6)	1993 (20.1)	2066 (20.0)	2138 (19.7)	2226 (19.4)	2209 (19.5)	2301 (20.3)
60–69 years	2240 (21.8)	2170 (21.8)	2116 (21.4)	2028 (20.2)	1977 (19.6)	2153 (20.6)	2082 (20.1)	2167 (20.0)	2437 (21.2)	2563 (22.6)	2506 (22.1)
70–79 years	2179 (21.2)	2180 (21.9)	2277 (23.0)	2434 (24.2)	2422 (24.0)	2616 (25.0)	2718 (26.3)	2698 (24.8)	2724 (23.7)	2591 (22.9)	2463 (21.7)
80–89 years	1179 (11.5)	1234 (12.4)	1155 (11.7)	1284 (12.8)	1262 (12.5)	1233 (11.8)	1291 (12.5)	1434 (13.2)	1501 (13.1)	1564 (13.8)	1625 (14.3)
90+ years	132 (1.3)	136 (1.4)	144 (1.5)	134 (1.3)	206 (2.0)	228 (2.2)	221 (2.1)	260 (2.4)	275 (2.4)	258 (2.4)	284 (2.5)
Localization											
Upper extremity	1515 (14.8)	1573 (15.8)	1518 (15.3)	1544 (15.4)	1589 (15.7)	1608 (15.4)	1650 (15.9)	1576 (14.5)	1735 (15.1)	1778 (15.7)	2002 (17.7)
Lower extremity	7553 (73.6)	7033 (70.8)	6993 (70.7)	7314 (72.8)	7373 (72.9)	7564 (72.4)	7475 (72.2)	7780 (71.6)	7867 (68.5)	8149 (71.9)	8371 (73.8)
Other region	1200 (11.7)	1326 (13.4)	1382 (14.0)	1195 (11.9)	1145 (11.3)	1280 (12.2)	1226 (11.8)	1504 (13.8)	1878 (16.4)	1404 (12.4)	967 (8.5)

## Discussion

In this population-based study trends in the epidemiology of osteomyelitis were described and prevalence was analyzed as a function of osteomyelitis subtype, anatomical localization, gender, and age group. Some literature provides insights regarding selected subgroups or subtypes of osteomyelitis, such as pediatric patients [19, 20] or vertebral osteomyelitis [21]. Additionally, the epidemiology of osteomyelitis has been analyzed based on a survey of residents of Olmsted County, Minnesota, United States, reporting 760 cases [22]. However, there is a lack of data estimating the prevalence of osteomyelitis for European countries. This study, to the best of our knowledge, is the first one describing the nationwide burden of osteomyelitis.

It was demonstrated that the number of cases increased by 10.44% over the last decade up to 16.7 per 100,000 inhabitants in 2018. In the light of a recent study calculating annual numbers of revision total knee arthroplasty procedures associated with periprosthetic joint infection (PJI) as 30.8 per 100,000 German inhabitants in 2018, forecasting an increase of almost 90% in 2050 [23], the dynamic in total osteomyelitis numbers seem surprisingly low. Here, the importance of delimitation between osteomyelitis and PJI becomes evident. Whereas the lines between the two diagnoses often appear to be blurred in literature [24–26], strictly applying the definition criteria of the European Bone and Joint Infection Society (EBJIS) [27] excludes PJI from the category ‘bone infection’. Further, PJI is distinctly coded according to the ICD-10 as “T84.5, infection and inflammatory reaction by a joint endoprosthesis” and therefore, not included in our analysis. Also, the prevalence rates in Germany were lower in comparison to 24.4 incident cases of osteomyelitis per 100,000 person-years estimated for the U. S [22], which might be explainable by differences in methodology. For instance, we did not consider vertebral osteomyelitis as spondylodiscitis is coded separately.

Our analysis also revealed that men were often affected than women, which is in accordance with findings by Kremers and colleagues reporting significantly lower incidences of 16.7 cases per 100,000 person-years for women compared to 27.7/100,000 for men [22]. Whereas underlying mechanisms are not fully understood yet, research increasingly addresses the importance of sex differences in immune response [28, 29]. The observed trend towards more osteomyelitis diagnosis in older patients possibly reflects demographic changes such as population decline and aging, which challenge the healthcare system not only in Germany. Further, higher numbers of chronic cases compared to acute cases were determined, whereas the prevalence of acute osteomyelitis rose by 61.8%. This finding may be attributable to recent advances in prevention strategies, early diagnosis of low-grade infections or awareness to discriminate acute and chronic bone infections [11, 30, 31]. However, potential biases in the distinction between acute and chronic osteomyelitis might influence these numbers as different definitions exits in the literature [32].

Our study is limited by the fact, that, although ICD-10 codes were available, it was not possible to differentiate possible driving comorbid factors, such as diabetes mellitus, peripheral vascular disease, trauma or the implantation of medical devices. Additionally, the analysed dataset did not include information regarding treatment procedure. Further, no statement regarding the distribution of pathogens causing the infection can be made. Also, we assumed correct diagnosing based on published criteria [31], however, a possible upcoding cannot be excluded.

## Conclusions

Osteomyelitis remains a serious problem for orthopedic and trauma surgery, also for countries comparable with Germany. In light of a strong increase especially in elderly patients, prevention strategies, improved treatment strategies and an interdisciplinary treatment approach are required.

## Data Availability

The datasets generated and analysed during the currents study are available from the corresponding author on reasonable request.
